# Mechanisms of microRNA Regulation of the Epithelial–Mesenchymal Transition (EMT) in Lung Cancer

**DOI:** 10.3390/life14111431

**Published:** 2024-11-06

**Authors:** Israel Martínez-Espinosa, José A. Serrato, Carlos Cabello-Gutiérrez, Ángeles Carlos-Reyes, Blanca Ortiz-Quintero

**Affiliations:** 1 Department of Molecular Biomedicine and Translational Research, Instituto Nacional de Enfermedades Respiratorias Ismael Cosío Villegas, Mexico City 14080, Mexico; 2 Department of Research in Virology and Mycology, Instituto Nacional de Enfermedades Respiratorias Ismael Cosío Villegas, Mexico City 14080, Mexico; 3 Laboratory of Onco-Immunobiology, Instituto Nacional de Enfermedades Respiratorias Ismael Cosío Villegas, Mexico City 14080, Mexico

**Keywords:** microRNAs, EMT, lung cancer

## Abstract

Lung cancer remains the cancer with the highest mortality worldwide, largely due to a limited understanding of the precise molecular mechanisms that drive its progression. microRNAs (miRNAs) have emerged as crucial regulators of lung cancer progression by influencing key cellular processes, notably the epithelial–mesenchymal transition (EMT). EMT is a complex and potentially reversible process where epithelial cells lose their polarity and adhesion, reorganize their cytoskeleton, and transition to a mesenchymal phenotype, enhancing their migratory and invasive capacities. While EMT plays an essential role in normal physiological contexts such as tissue development and wound healing, it is also a critical mechanism underlying the progression and metastasis of lung cancer. This review aims to summarize the latest research findings on the role of endogenous and exosome-derived microRNAs in regulating EMT in lung cancer, focusing on studies conducted over the past five years. It also provides an overview of EMT’s essential molecular mechanisms to better understand how miRNAs regulate EMT in lung cancer.

## 1. Introduction

Lung cancer has the highest mortality rate worldwide, with 2.2 million new cases and 1.8 million deaths estimated in 2020 [1]. The high mortality of lung cancer is attributable to late diagnosis and the presence of metastasis when treatment options are limited [2,3]. Epithelial-to-mesenchymal transition (EMT) is a critical mechanism driving cancer progression and metastasis, particularly in lung cancer. During EMT, epithelial cells lose their distinct characteristics and adopt features of mesenchymal cells. This transformation enhances their motility and invasiveness, essential for effective metastatic spread [4,5]. Lung cancer’s high rate of metastasis and mortality has been linked to the frequent occurrence of EMT in lung tumors. EMT contributes not only to the spread of cancer but also to drug resistance, both of which are significant challenges in effectively treating lung cancer [6,7]. Evidence shows that EMT is highly prevalent in advanced lung cancer, with approximately 60–70% of the cases of non-small cell lung cancer (NSCLC) displaying EMT markers. This prevalence is associated with poorer patient outcomes and a greater likelihood of cancer spread compared to other cancer types [6,8,9,10].

Recent studies highlight the crucial role of microRNAs (miRNAs) in regulating EMT, particularly in lung cancer. miRNAs are small RNAs that epigenetically regulate a wide range of essential biological processes by inhibiting target mRNAs in normal and disease conditions [5,11]. miRNAs regulate EMT by targeting the genes and pathways associated with EMT induction factors, the expression of adhesion molecules, motility, and enzyme production. Over the past five years, researchers have concentrated on identifying specific miRNAs that either promote or inhibit EMT in lung cancer. These findings reveal a complex network of interactions among the miRNAs, transcription factors, and signaling pathways involved in EMT. For instance, miRNAs, such as miR-4739 and miR-410, induce EMT-related pathways, including the Wnt/β-catenin, TGF-β, and PI3K/mTOR pathways, influencing EMT-promoting cancer properties [12,13]. Other miRNAs, such as miR-889-3p and miR-203, inhibit EMT-related pathways; however, they are often downregulated in lung cancer cells, ultimately promoting EMT [14,15]. Additionally, specific miRNAs often regulate lung cancer cells’ proliferation, migration, and invasion capacities, along with EMT, revealing a cooperative network effect. More importantly, research demonstrates that specific miRNAs regulating EMT significantly drive the progression of lung cancer cells in this disease. For instance, miR-4739 promotes the formation of pulmonary nodules, while miR-106a facilitates metastasis to the bone [12,16]. This evidence underscores the importance of miRNA regulation in EMT as a critical factor in lung cancer progression, highlighting potential targets for therapeutic intervention.

This review aims to summarize the latest research findings on the role of endogenous and exosome-derived microRNAs in regulating epithelial-to-mesenchymal transition (EMT) in lung cancer, focusing on studies conducted over the past five years.

## 2. microRNAs

microRNAs (miRNAs) are non-protein-coding RNA sequences measuring around 22 nucleotides in length. They function as the master regulators of gene expression at the post-transcriptional level by impeding the translation of specific messenger RNAs (mRNAs). miRNAs regulate multiple cellular processes critical for normal cellular functions, including cell cycle, cell differentiation, apoptosis, metabolism, immune response, and angiogenesis. In pathological conditions, miRNAs show different expression patterns that can be associated with disease development and progression [5,11,17,18,19]. In the context of cancer, miRNAs can function as oncomiRs or tumor suppressors, either promoting or inhibiting tumor progression. In lung cancer, miRNAs influence various biological processes crucial for tumor growth and metastasis, such as cell proliferation, migration, invasion, angiogenesis, immune response evasion, and EMT.

Most miRNAs surge from a canonical biogenesis pathway [11]. In the nucleus, genes encoding the miRNAs are transcribed by the RNA polymerase II (Pol II) into long primary precursors (pri-miRNAs) [20,21]. The microprocessor complex formed by ribonuclease Drosha and two molecules of the RNA-binding protein DiGeorge syndrome critical region 8 (DGCR8) cleaves this precursor to produce a smaller precursor called pre-microRNA, which is exported to the cytoplasm via Exportin-5 [22]. In the cytoplasm, the pre-miRNA is cleaved by the endonuclease Dicer/RNA-binding protein partners TRBP complex to produce mature double-stranded miRNAs with a length of 22 nucleotides [23]. Subsequently, the mature double-stranded miRNA is recruited by the RNA-induced silencing complex (RISC) formed by argonaute 1-2 (AGO1-2), Dicer/TRBP, and other proteins [24]. The double strand is separated, and one is retained on the RISC (guide strand) while the other is discarded (passenger strand). The miRNA guide strand binds to a partially complementary sequence in the target mRNA’s 3′ untranslated region (UTR) [25]. This binding leads to the inhibition of mRNA transcription through mRNA decay (mRNA destabilization) and translational repression [25,26].

These miRNAs are called “endogenous”, differentiating them from those the cells release within extracellular vesicles (EVs). EVs are lipid bilayer-bound particles that are secreted by nearly all cell types. They play a crucial role in intercellular communication. These vesicles, which include exosomes, microvesicles, and apoptotic bodies, vary in size, biogenesis, and cargo, highlighting their distinct roles in biological processes. Each cell type releases EVs with distinct features that reflect how the cell functions. For example, immune cells, like lymphocytes and dendritic cells, release EVs that help regulate immune responses. On the other hand, cancer cells release EVs that are enriched with oncogenic proteins, miRNAs, and other signaling molecules that support tumor progression by facilitating processes such as angiogenesis, immune evasion, and metastasis. Emerging research highlights the critical role of EVs in lung cancer, especially those that contain miRNAs, which include exosomes and microvesicles. However, most lung cancer studies have concentrated on exosomes [27,28]. The formation of exosomes involves the inward budding of the cytoplasmic membrane to create early endosomes, which mature into late endosomes. Multiple intraluminal vesicles (ILVs) are formed within them, creating multivesicular bodies (MVBs). These ILVs contain specific cargoes such as miRNAs, proteins, nucleic acids, noncoding RNAs, glycoproteins, and lipids [29]. MVBs can fuse with the plasma membrane to release their ILV contents as exosomes into the extracellular space. These miRNAs and the other molecules are transported as cargo in exosomes and released to the extracellular space, where recipient cells can take them up and deliver their cargo. The miRNAs carried by EVs/exosomes can modulate gene expression in the recipient cell by binding to complementary sequences in target mRNAs, leading to mRNA decay or the inhibition of translation. Recipient cells take up exosomes through various mechanisms, including receptor binding, endocytosis, micropinocytosis, phagocytosis, and direct membrane fusion [17,30,31].

## 3. The EMT

**Figure 1:**
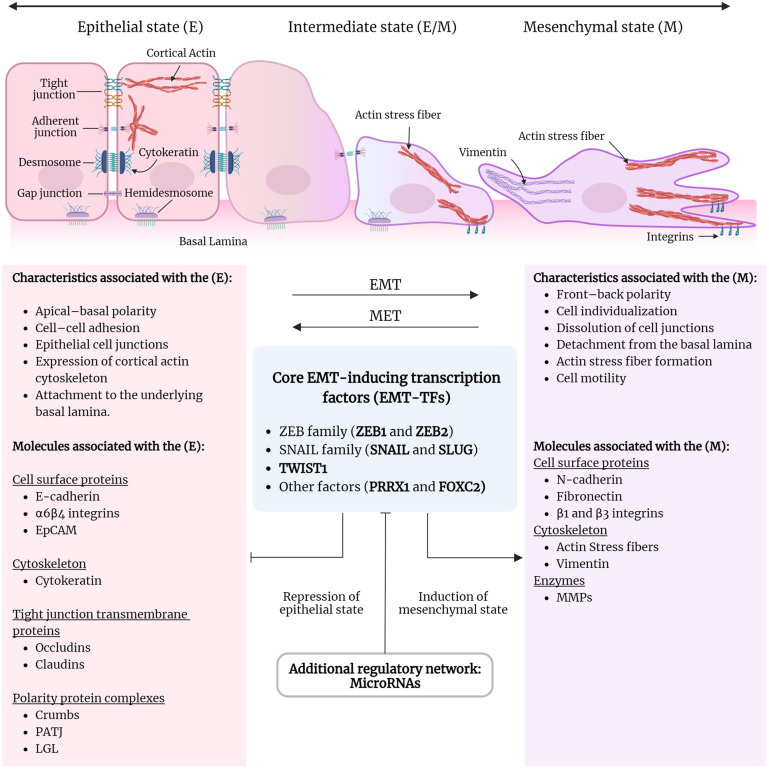
The figure illustrates the critical cellular and molecular events that occur during EMT. During EMT, epithelial cells lose their distinctive characteristics and adopt features typical of mesenchymal cells. This process includes several intermediate states between the epithelial and mesenchymal forms. Mesenchymal cells can transition to the epithelial state through a process known as mesenchymal–epithelial transition (MET). Epithelial cells are linked by tight junctions, adherens junctions, desmosomes, and gap junctions, while hemidesmosomes anchor them to the basal lamina, maintaining their apical–basal polarity. Mesenchymal cells display front–back polarity, detach from one another and the basal lamina, gain motility, and have the capacity to break down the extracellular matrix (ECM). Specific proteins found on cell surfaces, cytoskeletal and cell–cell junction proteins, and ECM-degrading enzymes such as matrix metalloproteinases (MMPs) often serve as biomarkers to identify each phenotype. Activating EMT transcription factors (EMT-TFs) like ZEB, SNAIL, and TWIST facilitates the acquisition of the mesenchymal state. Additionally, a regulatory network that includes microRNAs and splicing factors modulates the EMT-TFs, enabling the transition between the mesenchymal and epithelial states. This figure was created using BioRender.com.

According to the EMT International Association (TEMTIA), EMT refers to a complex and often reversible change in cellular characteristics. Epithelial cells undergo EMT, a well-conserved program enabling these cells to assume mesenchymal properties under various physiological and pathological conditions. This dynamic, multistage process is vital in development, tissue repair, wound healing, and cancer progression, where it provides cells with enhanced migratory and invasive abilities crucial for adapting to diverse cellular environments. During EMT, epithelial cells lose their apical–basal polarity, change their cytoskeleton, and display reduced adhesion to other cells [32]. Typically, epithelial cells exhibit dual polarity, including apical–basal polarity and planar polarity, and connect with other cells through junctions like desmosomes, tight junctions, adherens junctions, and gap junctions. In contrast, mesenchymal cells exhibit front–back polarity, detach from other cells and the basal lamina, gain motility, and degrade the extracellular matrix (ECM). The recent literature describes the EMT and the MET as highly dynamic and context-dependent processes essential for cellular plasticity. These transitions are not linear; instead, they involve intermediate states, allowing cells to exhibit hybrid phenotypes that combine epithelial and mesenchymal characteristics. This plasticity enables cells to adapt to environmental cues and supports a reversible transition model influenced by factors such as inflammation and growth signals, particularly in cancer metastasis [32,33,34]. The EMT induces changes in molecular and cellular events that involve activating core EMT-inducing transcription factors (EMT-TFs), including the ZEB family (ZEB1 and ZEB2), the SNAIL family (SNAIL1 and SNAIL2, also known as SNAIL and SLUG), and TWIST1. This activation leads to the disruption of cell–cell junctions, inhibition of epithelial proteins, and induction of mesenchymal-specific cell surface proteins, the reorganization and expression of cytoskeletal proteins, and the production of ECM-degrading enzymes such as matrix metalloproteinases (MMPs). An additional regulatory network, which includes miRNAs and splicing factors, controls the EMT-TFs to switch between mesenchymal and epithelial states [32,33,34]. Figure 1 represents the critical cellular and molecular events during EMT.

The stromal microenvironment emits several signals that activate EMT. These signals and their signaling pathways are often interconnected in complex networks that regulate EMT. The main pathways known to trigger EMT in normal and pathological conditions are the transforming growth factor-β (TGF-β) pathway, the Wnt signaling pathway, the Notch signaling pathway, and mitogenic growth factors such as the epidermal growth factor (EGF), fibroblast growth factor (FGF), and inflammatory cytokines. These triggering pathways converge to activate the core EMT-TFs and induce EMT. In cancer, the tumor microenvironment’s stromal cells also contribute to the EMT activation of cancer cells by secreting several pro-EMT cytokines. For example, cancer-associated fibroblasts (CAFs) secrete TGFβ, IL-6, and vascular endothelial growth factor alpha (VEGFA) [35,36,37]. Tumor-associated macrophages (TAMs) secrete TGFβ and tumor necrosis factor alpha (TNFα), which synergize with TGFβ to promote EMT [38,39].

### 3.1. The EMT in Wound Healing and Development

The EMT and MET are essential in wound healing by reorganizing epithelial cells and their interactions with blood vessels, stroma, and surrounding tissues. After an injury, epithelial cells at the wound’s edge undergo EMT, enabling them to migrate, cover the wound area, and interact with nearby vascular and stromal components. This supports tissue repair. Cells that undergo EMT release substances that promote the formation of new blood vessels, essential for healing. They also produce matrix metalloproteinases (MMPs), enzymes that reshape the extracellular matrix (ECM), creating a suitable environment for cell migration and tissue formation. These changes impact nearby cells, such as fibroblasts and mesenchymal stem cells, which assist in healing. Once the wound is nearly closed, MET occurs, restoring the surface layer of cells [40,41].

In addition, EMT is essential in several stages of embryonic development, including gastrulation, neural crest cell formation, and organ development [41,42,43]. During early development, EMT facilitates primary cell migrations, leading to the formation of germ layers. In gastrulation, cells move inward to generate mesodermal tissues and structures. When forming neural crest cells, they undergo EMT to detach from the neuroepithelium and migrate to different regions of the developing embryo. This migration is essential for forming diverse tissues, including craniofacial cartilage and neurons [43]. EMT controls migratory cell ability, vital for developing the complex structures and cell types that comprise the vertebrate body [41,42]. EMT also plays a significant role in organogenesis, which is the formation of essential organs such as the heart and kidneys. During heart development, epicardial cells undergo EMT to differentiate into epicardial-derived cells. These cells contribute to various cardiac tissues, including the myocardium and coronary vessels [44]. Likewise, EMT organizes mesenchymal cells in kidney formation into nephron units, which are essential for kidney filtration functions [45].

### 3.2. The EMT in Cancer 

The association between EMT and invasion, metastasis, and cancer progression has been well documented in various types of cancer. EMT is believed to be a driver of cancer progression. However, whether EMT is a causal factor or an essential requirement is still under investigation. Several studies have shown the relevant role of EMT in the spread of cancer cells and their ability to form metastases. For example, in a mouse model of squamous cell carcinoma (SCC), researchers discovered that TWIST1 can induce EMT and enable tumor cells to spread into the bloodstream [46]. Similarly, reducing the expression of ZEB1 significantly, but partially, inhibited metastasis in a mouse model of pancreatic cancer [47]. In breast cancer models, inhibiting SNAIL1 or TWIST reduces tumor metastasis in vivo [48,49]. Although further experimental investigations are ongoing to confirm the necessity of EMT in invasion and metastasis, the evidence does indicate that EMT provides cancer cells with several characteristics associated with invasive and metastatic tumors. Therefore, EMT is a critical participant in the malignant progression of cancer. 

**Figure 2:**
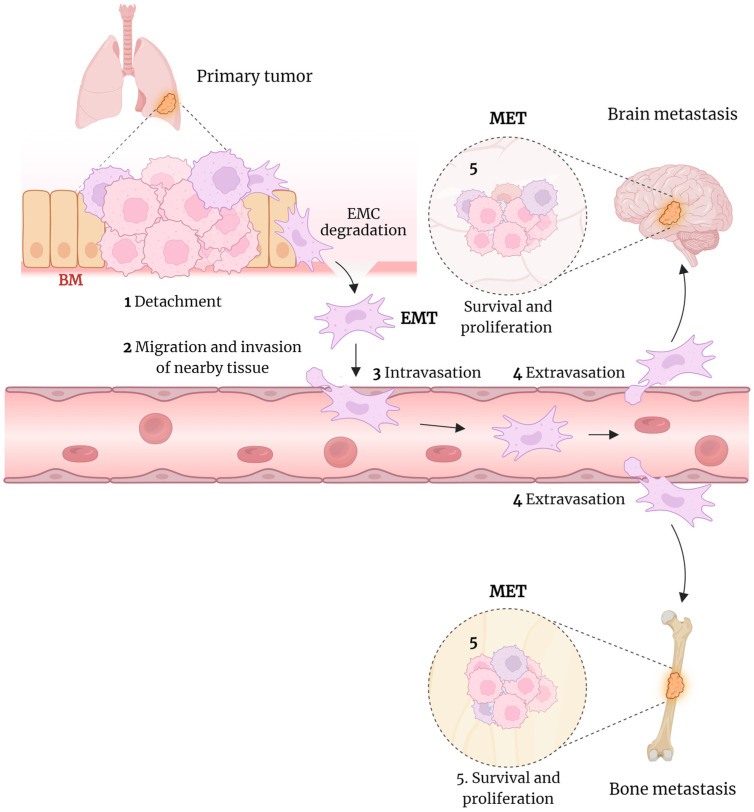
The role of the EMT in lung cancer progression. EMT allows lung cancer to become mobile, break down barriers, and migrate to other tissues. Once in a new environment, the cancer cells must adapt, survive, and revert to an epithelial state, allowing them to proliferate and establish a new metastatic cancer site. A more detailed description is provided in the main body of the text. This figure was created using BioRender.com.

The role of EMT in cancer is to enable cancer cells to become mobile, break down the basement membrane (BM) and extracellular matrix (ECM), and migrate and invade nearby tissues. These cancer cells can then enter the systemic circulation, where they must withstand flow-induced stress, immune response, anoikis, and oxidative stress [49,50,51,52]. The surviving cancer cells can become trapped in capillaries and exit into other organs’ parenchyma by crossing endothelial cells. Once in a new environment, the cancer cells must adapt and survive before transitioning to an epithelial state through mesenchymal–epithelial transition (MET). This transition allows them to proliferate and establish new metastatic cancer sites, such as the brain or bones in lung cancer [52] (Figure 2).

Furthermore, EMT has been associated with therapy resistance in cancer, including lung cancer, which may contribute to recurrence and cancer progression [7,53]. Various mechanisms that may cause the therapy resistance associated with EMT have been described [54,55,56]. For instance, EMT induces resistance to tyrosine kinase inhibitor (TKI) therapies in lung cancer by activating the SNAIL1-mediated overexpression of AXL. This activation enhances the signaling of the PI3K/Akt pathway, which is typically inhibited by EGFR-targeted TKIs. This finding supports the development of combined therapeutic strategies that target both EGFR and AXL or the compensatory signaling pathways of PI3K/Akt. Such approaches offer an alternative way to overcome TKI resistance in lung cancer [7,57]. Another mechanism involves the EMT inducing the downregulation of the components of the pro-apoptotic pathways. For example, Slug, another EMT-TF, inhibits the expression of the pro-apoptotic gene PUMA, promoting cisplatin resistance in NSCLC cells [58]. In addition, EMT phenotype markers have been associated with the poor prognosis and prediction of therapy resistance in lung cancer, indicating its potential clinical relevance [7,53,59,60].

## 4. microRNAs That Regulate EMT in Lung Cancer

The latest research indicates that both endogenous miRNAs and those carried by EVs play significant roles in advancing lung cancer by regulating the EMT process. Specific miRNAs can promote or inhibit EMT in lung cancer depending on the target gene and the cellular and environmental context. In general, miRNAs that target tumor suppressor genes and promote tumor progression are called oncomiRs. Conversely, miRNAs that target oncogenes and suppress tumor progression are known as tumor suppressor (TS) miRNAs [61,62]. Those miRNAs acting as oncomiRs are typically upregulated in cancer cells, while miRNAs acting as TS miRNAs are generally downregulated. Therefore, the observed effect promotes EMT and cancer progression.

The following sections detail the miRNAs involved in regulating EMT in lung cancer based on studies conducted over the last five years.

### 4.1. Endogenous miRNAs Promoting EMT in Lung Cancer

**Figure 3:**
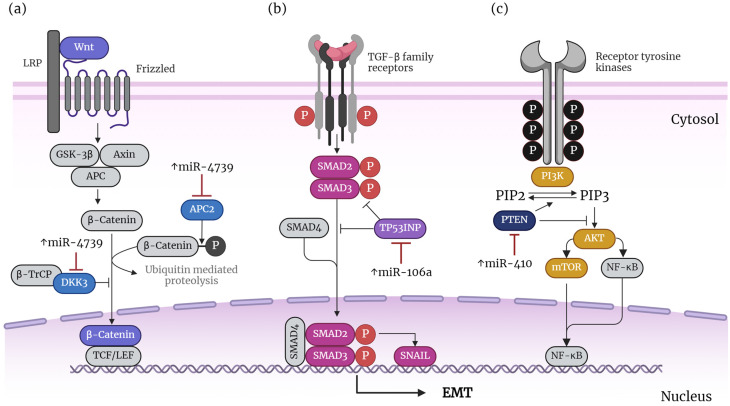
Schematic representation of how endogenous miRNAs regulate the promotion of EMT in lung cancer. The diagram depicts a potential regulatory mechanism based on evidence from the reviewed studies, shown in blue, purple, magenta, dark blue, and gold. The identified factors in those colors are assembled in a hypothetical pathway, drawn in gray, based on well-known EMT-promoting pathways and information from the independent studies of other cancers. (**a**) miR-473, (**b**) miR-106a, and (**c**) miR-410. ↑ indicates upregulation. A more detailed description is provided in the main body of the text. This figure was created using BioRender.com.

A study found that miR-4739 was upregulated in NSCLC tumor tissues that did not have specific drug-sensitive mutations like EGFR, ALK, and ROS1 gene mutations (“driver negative”). Notably, the increase in miR-4739 was independently linked to poor overall survival. The overexpression of miR-4739 caused driver-negative NSCLC cell lines (H2085 and H2126) to undergo EMT, proliferate, and migrate. In vivo experiments also showed that miR-4739 led to larger subcutaneous tumors and more metastatic tumors in the lungs. This effect was achieved by miR-4739 by inhibiting the APC regulator of WNT signaling pathway 2 (APC2) and Dickkopf WNT signaling pathway inhibitor 3 (DKK3), which are known antagonists of the Wnt/β-catenin pathway, leading to its activation [12] (Table 1 and Figure 3a). Recently, another study revealed that miR-106a is overexpressed in lung adenocarcinoma (AD) tissue in patients with brain metastasis (BM). miR-106a promotes cell proliferation, migration, invasion, and EMT while inhibiting apoptosis and autophagy-dependent cell death. These effects are achieved by targeting the tumor protein p53-inducible nuclear protein 1 (TP53INP1). Research showed that miR-106a increases the phosphorylation levels of SMAD/2, which is known to induce EMT in the TGF β pathway. At the same time, the upregulation of TP53INP1 reverses the effect, indicating the miR-106a/TP53INP1 regulation of EMT. Importantly, miR-106a promotes metastasis in vivo, particularly to the bones, showing its relevance in metastasis [16] (Table 1 and Figure 3b). Another miRNA that triggers EMT is miR-410. miR-410 is elevated in several NSCLC cell lines, and its overexpression resulted in EMT, resistance to radiation, and improved DNA damage repair. miR-140 suppresses its target, the phosphatase and tensin homolog (PTEN). This study found that the overexpression of PTEN and using inhibitors for the PI3K, Akt, and mTOR pathways can reverse the effects of miR-410. This suggests that miR-410 inhibits PTEN, which counteracts the PI3K/mTOR pathway to induce EMT [13] (Table 1). It is known that PTEN is a lipid phosphatase that negatively regulates the PI3K/mTOR pathway through the dephosphorylating of the dephosphorylation of phosphatidylinositol (3,4,5)-trisphosphate (PIP3) to phosphatidylinositol (4,5)-bisphosphate (PIP2) (see Figure 3c) [63].

These studies show endogenous miRNAs promote EMT by regulating the key components of well-known EMT-related pathways, such as the Wnt/β-catenin, TGF-β, and PI3K/mTOR pathways (see Figure 3). Furthermore, the regulation of specific miRNAs associated with EMT enhances the ability of lung cancer cells to progress in the disease. For instance, miR-4739 promotes the formation of pulmonary nodules, while miR-106a facilitates metastasis to the bone. miR-4739 achieves this notable effect by effectively inhibiting two molecular factors that hinder the WNT signaling pathway: APC2 and DKK3. Both APC2 and DKK3 are well-known antagonists of the Wnt/β-catenin pathway. By suppressing these inhibitors, miR-4739 facilitates the activation of this signaling route, leading to significant downstream biological effects, such as increased EMT, proliferation, and migration of lung cancer cells. Although the molecular mechanisms were not verified experimentally in this study, it is known that the cytoplasmic form of DKK3 (DKK3-b) forms a complex with β-TrCP and impairs the nuclear translocation of β-catenin, inhibiting Wnt/β-catenin signaling, as illustrated in Figure 3a. In addition, APC2 recruits and phosphorylates the Wnt effector beta-catenin and targets beta-catenin for ubiquitylation and proteasomal degradation, also illustrated in Figure 3a. Meanwhile, miR-106a suppresses TP53INP1, which prevents the phosphorylation of Smad2/3. As shown in Figure 3b, Smad2/3 are known components of the TGF-β pathway. Consequently, miR-106a enhances the activation of this pathway, resulting in increased EMT and migration of lung cancer cells. Notably, although the crucial role of miR-106a in promoting metastasis to the bone was established, the participation of TP53INP1/Smad2/3 still requires experimental examination in this study.

This evidence underscores the importance of miRNA regulation in EMT as a critical factor in lung cancer progression, highlighting potential targets for therapeutic intervention.

### 4.2. Endogenous miRNAs Suppressing EMT in Lung Cancer

**Figure 4:**
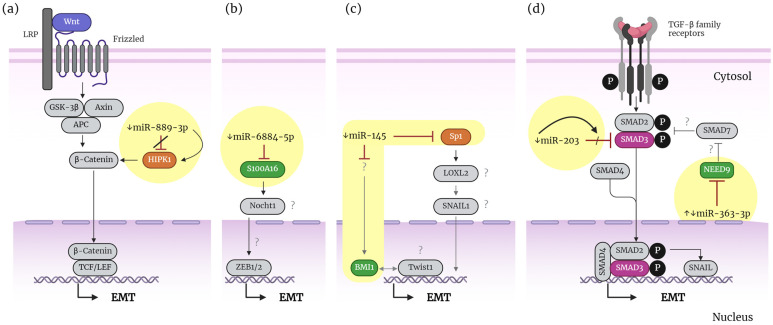
Schematic representation of how endogenous miRNAs regulate the inhibition of EMT in lung cancer. The diagram depicts a potential regulatory mechanism based on evidence from the reviewed studies, highlighted in yellow circles and bright colors. The identified factors in bright colors are assembled in a hypothetical pathway, drawn in gray, and based on information from the independent studies of other cancers. (**a**) miR-889-3p. (**b**) miR-6884-5p. (**c**) miR-145. (**d**) miR-203 and 363-5p.↓ indicates downregulation. ↑ indicates upregulation. (/) indicates blocking. (?) indicates an unknown potential factor or component of the pathway. A more detailed description is provided in the main body of the text. This figure was created using BioRender.com.

A recent study found reduced expression of miR-6884-5p in NSCLC tissues. This reduction correlated with increased tumor invasion and later TNM stage but showed no association with age and gender. The induced overexpression of miR-6884-5p inhibits EMT in lung adenocarcinoma (AD) A549 cells via targeting the S100 calcium-binding protein A16 (S100A16) [64]. S100A16 has been linked to EMT regulation in breast cancer and pancreatic ductal adenocarcinoma (PDA) through Notch1 pathways and FGF19/AKT activation, respectively [65,66] (Table 1). However, the current study did not further investigate the mechanisms involved in miR-6884-5p/S100A16 regulation (Figure 4b). Another recent study found that miR-503 levels were low in gefitinib-resistant cells. These drug-resistant cells exhibited increased abilities for EMT, migration, and invasion. However, the induced overexpression of miR-503 did not impact drug resistance but inhibited those cells’ EMT, migration, and invasion capabilities. The study also identified the protein tyrosine kinase 7 (PTK7) as the target gene miR-503. The overexpression of PTK7 led to increased migration and invasion through focal adhesion kinase (FAK) and paxillin activation, but it did not affect the EMT. Therefore, the target genes and pathways involved in the regulation of EMT by miR-503 were not identified in this study [67] (Table 1). Researchers discovered a reduced expression of miR-145-5p in pemetrexed-resistant cells derived from A549 cells, known as A400 cells. By increasing the levels of miR-145-5p, the sensitivity of A400 cells to pemetrexed is enhanced, leading to a reduction in proliferation and EMT. This is achieved by targeting specificity protein 1 (Sp1) by miR-145-5p (Table 1). The study also revealed that miR-145-5p suppresses the expression of the BMI1 proto-oncogene polycomb ring finger (BMI1) indirectly, which promotes proliferation and EMT in NSCLC cells [68]. Sp1 is a zinc finger transcription factor that binds the GC-rich motifs of many promoters, and it was linked to EMT regulation through lysyl oxidase-like 2(LOXL2) in PDA [69]. BMI1 was found to be regulated by Twist1 to promote EMT in head and neck squamous cell carcinoma (HNSCC) [70]. However, the current study did not uncover the mechanistic connection involving miR-145-5p/Sp1/EMT or miR-145-5p/BMI1/EMT (Figure 4c).

On the other hand, miR-889-3p was found to be reduced in lung cancer tissues and cell lines. miR-889-3p inhibits the proliferation, invasion, and EMT of A549 cells by targeting the homeodomain-interacting protein kinase 1 (HIPK1). Additionally, the induced overexpression of miR-889-3p and HIPK1 knockdown hinders subcutaneous tumor growth in vivo, suggesting a relevant role in cancer progression [14] (Table 1). HIPK1 is a serine/threonine-protein kinase found to phosphorylate β-catenin on Ser552, stabilizing and activating it in breast cancer stem cells [71]. However, this study did not explore the mechanisms involved in miR-889-3p/HIPK1 (Figure 4a). In a separate study, researchers found that the 95D cell line exhibited the lowest level of miR-188-5p expression compared to other NSCLC cell lines. Then, they overexpressed miR-188-5p in this cell line and found that it inhibits EMT, migration, and invasion. miR-188-5p targets directly the beta-1,4-Mannosyl-Glycoprotein 4-Beta-N-Acetylglucosaminyltransferase (MGAT3) but indirectly reduces the expressions of the EMT-TF Snail. However, no further details regarding the mechanisms involved were explored [72] (Table 1). Another study found that miR-363-3p overexpression reduced EMT, migration, invasion, MMP2, and MMP9 levels in H1299 cells. Conversely, the inhibition of miR-363-3p had the opposite effect in A549 cells. miR-363-3p targets the neural precursor cell expressed, developmentally downregulated 9 (NEDD9), and the SRY-box transcription factor 4 (SOX4). Overexpressing NEDD9 or SOX4 reversed the effects of miR-363-3p, indicating their involvement in regulating EMT, migration, and invasion (Table 1). Significantly, the overexpression of miR-363-3p induces the in vivo inhibition of lung metastasis, demonstrating its repressive effect in vivo. However, the study did not explore the mechanisms of the miR-363-3p/NEDD9/SOX4 regulation of EMT further [73] (Figure 4d). A potential mechanism may involve NEDD9 inhibiting Smad7, such as that described in hepatocellular carcinoma cells [74]. It is known that Smad7 regulates the TGF-β1 signal pathway by inhibiting the phosphorylation of Smad2 and Smad3 [75] (Figure 4d). Recent findings showed that miR-203, downregulated in 10 NSCLC tissues, reduces the TGF-β-induced EMT, migration, and invasion of the H226 cell line when transfected with miR-203 mimics. The study identified SMAD3 as a target for miR-203, which is known to activate EMT via the TGF-β pathway. Still, it did not provide further experimental evidence regarding the mechanism [15] (Table 1). An independent study found that miR-146b is downregulated in cisplatin-resistant (CR) AD cells (A549 and H1299). Overexpressing miR-146b inhibits EMT and cell viability in CR cells in vitro. Researchers discovered that miR-146b targets the protein tyrosine phosphatase 1B (PTP1B) and regulates cell viability. However, they did not provide evidence of regulating EMT via PTP1B. In vivo experiments showed that the overexpression of miR-146b suppressed the growth of CR cells in mice and inhibited the EMT phenotype (Table 1). No further pathway insights were explored [76].

Together, these studies show that several endogenous miRNAs inhibit EMT in lung cancer. However, these miRNAs are often downregulated in lung cancer cells, ultimately promoting EMT. Despite these findings, many studies provide limited information about the pathways or molecular factors involved in this regulation. Most identify the target gene for tumor-suppressor miRNAs and their impact on EMT but do not further investigate the underlying molecular mechanisms (see Figure 4). Additionally, some studies lack sufficient experimental evidence to substantiate the regulation of EMT by the identified miRNA targets (Figure 4). On the other hand, tumor-suppressor miRNAs, such as miR-363-3p and miR-889-3p, have been shown to inhibit lung metastasis and tumor growth in vivo, underscoring their significance in cancer progression. However, further research is needed to accurately determine the mechanisms by which TS miRNAs regulate EMT in these contexts. Table 1 summarizes the endogenous miRNAs associated with the regulation of EMT in lung cancer based on studies from the last five years.

**Table 1:** Endogenous miRNAs that regulate EMT in lung cancer.

miRNA	Source	Expression	miRNA Gene Target	Pathway	Effect	Reference
miR-4739	NSCLC tumor tissues (“driver negative”)	Up	APC2 and DKK3	Wnt/β-catenin pathway activation.	Promotion of EMT, proliferation, and migration in H2085 and H2126 cells. Increase metastatic pulmonary nodules.	[12]
miR-106a	AD tissue of a patient with BM	Up	TP53INP1	Phosphorylation of smad2/3	Promotion of EMT, migration, autophagy-dependent cell death, and metastasis to bone.	[16]
miR-410	NSCLC cell lines	Up	PTEN	PI3K/mTOR pathway activation	Promotion of EMT and radioresistance.	[13]
miR-6884-5p	NSCLC tissues	Down	S100A16	Not explored	Inhibition of the EMT of A549 cells.	[64]
miR-503	Gefitinib-resistant HCC827	Down	Unknown for EMT	Unknown for EMT	Inhibition of EMT, migration, and invasion.	[67]
miR-145-5p	Pemetrexed-resistant A400 cells	Down	Sp1	Not explored	Inhibition of EMT and increased sensitivity to pemetrexed.	[68]
miR-889-3p	Lung Cancer tissue and cell lines	Down	HIPK1	Not explored	Inhibition of the EMT, proliferation, and invasion of A549 cells, and subcutaneous tumor growth in vivo.	[14]
miR-188-5p	95D cell line	Down	MGAT3	Indirect reduction in Snail expression	Inhibition of the EMT, migration, and invasion of 95D cells.	[72]
miR-363-3p	NSCLC cell lines	Varies	NEDD9 and SOX4	Not explored	Inhibition of EMT, migration, and invasion. Inhibition of lung metastasis in vivo.	[73]
miR-203	NSCLC tissue	Down	SMAD3 *	Not explored	Inhibition of the TGF-β-induced EMT, migration, and invasion of the H226 cell line	[15]
miR-146b	Cisplatin-resistant (CR) AD cells	Down	PTP1B *	Not explored	Inhibition of EMT and cell viability of CR cells.	[76]

AD, lung adenocarcinoma; APC2, APC regulator of WNT signaling pathway 2. DKK3, Dickkopf WNT signaling pathway inhibitor 3. * there is no experimental evidence to support the regulation of EMT via this target in this study.

### 4.3. Exosomal miRNAs Promoting EMT in Lung Cancer

Several tumor-derived exosome miRNAs have been shown to regulate EMT by promoting or inhibiting it in the recipient cell. Exosomal miR-31-5p, miR-210-3p, and miR-499a-5p are among those that promote EMT in lung cancer.

**Figure 5:**
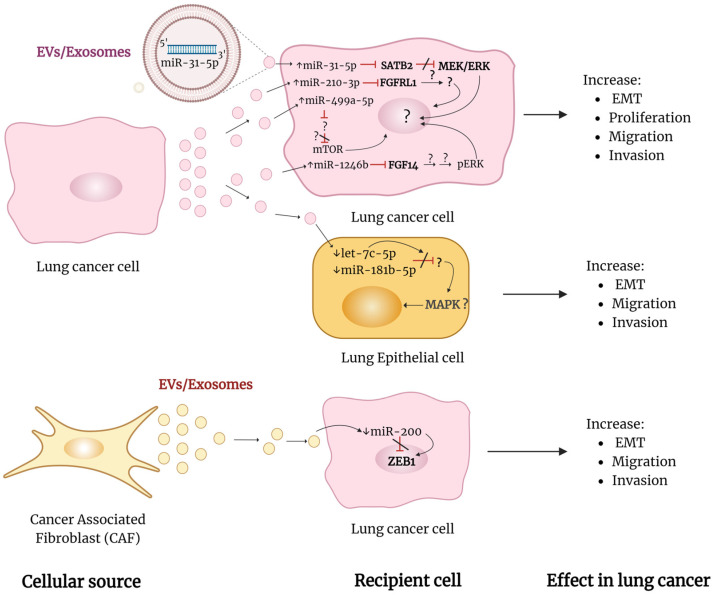
Schematic representation of how exosomal miRNAs regulate EMT in lung cancer. The diagram depicts the evidence reported in the reviewed studies. Lung cancer cells release vesicles (EVs/exosomes) that transport miRNAs to other cancer cells or normal cells. Inside the recipient cell, miRNAs bind to their mRNA targets and regulate EMT, migration, and invasion. Other components of the tumor microenvironment, such as CAFs, also contribute to EMT regulation by releasing EVs/exosomes carrying miRNAs that affect target genes associated with EMT in recipient cells. ↓ indicates downregulation. ↑ indicates upregulation. (/) indicates blocking. EVs, extracellular vesicles. SATB2, special AT-rich sequence-binding protein 2. FGFRL1, fibroblast growth factor receptor-like 1. FGF14, fibroblast growth factor 14. ZEB1, zinc finger E-box-binding homeobox 1. ERK, extracellular signal-regulated kinase. MEK, mitogen-activated protein kinase. mTOR, mammalian target of rapamycin. A more detailed description is provided in the main body of the text. This figure was created using BioRen-der.com.

During hypoxic conditions, A549 and H1299 lung cancer cells produce exosomes enriched with miR-31-5p. Exosomal miR-31-5p derived from these hypoxic cells promotes the EMT, migration, invasion, and activation of the MEK/ERK pathway in the same cells when cultured under normal oxygen conditions. This contributes to lung tumor metastasis both in vitro and in vivo. The exosomal miR-31-5p achieved this effect by targeting the special AT-rich sequence-binding protein 2 (SATB2), repressing its expression. In this study, silencing SATB2 enhances the EMT and increases the phosphorylation of MEK and ERK [77] (Table 2). SATB2 is known to act as a negative regulator of EMT by downregulating transcription factors such as Snail, Slug, Zeb1, and Zeb2, although this was not examined in the current study (Figure 5). Also, it was found to reduce the activity of MEK/ERK in NSCLC and colorectal cancer [78,79].

Cancer stem cells (CSCs) derived from A549 cells produce exosomes enriched with miR-210-3p. Exosomal miR-210-3p from these lung CSCs enhanced the EMT phenotype and migratory and invasive abilities of A549 and NCI-H1703 cells by silencing the fibroblast growth factor receptor-like 1 (FGFRL1). This study did not further examine the precise molecular mechanism involved [80] (Table 2 and Figure 5). In a different study, lung cancer cells resistant to osimertinib, HCC827-OR, transfer exosomal miR-210-3p, inducing EMT and osimertinib resistance in the parental cells HCC827 [81]. Similarly, this study did not investigate the underlying molecular mechanisms involved (Table 2).

The SPC-A-1BM cell line, a highly metastatic lung adenocarcinoma cancer cell, produces exosomes enriched with miR-499a-5p. These miR-499a-5p-enriched exosomes increase the EMT, proliferation, and migration of the parental SPC-A-1 cell line. Experiments involving the overexpression of miR-499a-5p showed that the increased EMT, proliferation, and migration of transfected cells were associated with increased levels of phosphorylated ribosomal protein S6 kinase B1 (S6K1) and eukaryotic translation initiation factor 4E binding protein 1 (BP1) proteins and the activation of the mTOR pathway. Additionally, miR-499a-5p induces larger tumor nodules in an intracutaneous mice model, suggesting its potential role in tumor progression (Table 2). The study noted that while the direct target gene of miR-499a-5p has yet to be investigated, potential interactions between the target gene and the mTOR pathway remain to be explored [82] (Figure 5). 

Recently, it was found that miR-1246b is upregulated in bronchoalveolar lavage fluid extracellular vesicles (BALF-EVs) from patients with malignant pulmonary nodules. By inhibiting the fibroblast growth factor 14 (FGF14), miR-1246b promotes the proliferation, migration, invasion, EMT, and ERK phosphorylation of lung cancer cells. Whether other proteins downstream of FGF14 also play a role in the ERK pathway has not been investigated. Xenograft experiments demonstrated that lung cancer cells overexpressing miR-1246b develop tumors with increased size and weight compared to the control group. This suggests a significant role in the progression of lung tumors [83] (Table 2).

### 4.4. Exosomal miRNAs Suppressing EMT in Lung Cancer

A study showed that levels of let-7c-5p and miR-181b-5p were decreased in exosomes derived from lung adenocarcinoma A549 cells. These exosomes promote the EMT, migration, and invasion of the epithelial lung normal cells BEAS-2B. When exosomes from A549 cells overexpressing let-7c-5p and miR-181b-5p were used, there was a decrease in migration and invasion properties on BEAS-2B cells, indicating an inhibitory effect. The bioinformatic analysis suggested that let-7c-5p and miR-181b-5p may regulate the mitogen-activated protein kinase (MAPK) signaling pathway, but this was not experimentally tested [84] (Table 2 and Figure 5). Another inhibitory miRNA transferred by exosomes is miR-200. Exosomes secreted by cancer-associated fibroblasts (CAFs) isolated from NSCLC tissue have lower miR-200 levels than those secreted by normal fibroblasts. CAFs are one of the most abundant stromal cells in the tumor microenvironment that promote tumor progression in lung cancer. Exosomes from CAFs transfected with miR-200 inhibit the migration, invasion, and EMT of A549 and NCI-H460 cells by repressing the zinc finger E-box-binding homeobox 1 (ZEB1), confirming the inhibitory effect [85] (Table 2 and Figure 5). 

### 4.5. Summary of Exosomal miRNAs Regulating EMT in Lung Cancer

The literature indicates that lung cancer cells release exosomal miRNAs that function as oncomiRs in recipient lung cancer cells, promoting EMT. They also release exosomal miRNAs that act as tumor suppressor (TS) miRNAs, which are downregulated in these exosomes. When normal recipient cells uptake these exosomes, they induce the opposite effect, thereby promoting EMT. Cancer-associated fibroblasts (CAFs), a significant component of the tumor microenvironment, also release exosomes containing miRNAs that promote EMT in recipient lung cancer cells, highlighting the importance of the tumor microenvironment in this process. However, the existing studies have limitations as they need to provide further mechanistic details regarding the regulation of EMT by exosomal miRNAs (see Figure 5). Despite these limitations, the research underscores the importance of exosome-transported miRNAs in lung cancer progression. For instance, exosomal miR-31-5p and EVs-miR-1246b have been shown to promote lung metastasis and increase tumor size in vivo.

As mentioned before, the evidence from in vivo studies underscores the notable relevance of exosomal miRNA regulation in EMT as a critical factor in lung cancer progression, highlighting potential targets for therapeutic intervention.

Table 2 summarizes the exosomal miRNAs associated with the regulation of EMT in lung cancer based on publications from the last five years. Figure 5 shows a schematic representation of how exosomal miRNAs regulate EMT in lung cancer from these recent publications.

**Table 2:** Exosomal miRNAs that regulate EMT in lung cancer.

miRNA	Source	miRNA Gene Target	Pathway	Function	Reference
Exosomal miR-31-5p	Hypoxic A549 and H1299 cells	SATB2	Increased MEK/ERK signaling activation	Promotion of the EMT, migration, and invasion of normoxic tumor cells, and lung metastasis in vivo.	[77]
Exosomal miR-210-3p	Lung-CSC-derived A549 cells	FGFRL1	Unknown	Promotion of the EMT, migration, invasion, and MMP-9/MMP-1 expression of A549 and H1703 cells.	[80]
Exosomal miR-210-3p	HCC827-OR cells	Unknown	Unknown	Promotion of the EMT and resistance to osimertinib of HCC827 parental cells.	[81]
Exosomal miR-499a-5p	SPC-A-1BM cells	Unknown	Increased mTOR pathway activation.	Promotion of the EMT, proliferation, and migration of SPC-A-1 parental cells.	[82]
EVs-miR-1246b	BALF from patients with malignant pulmonary nodules	FGF14	Increased ERK phosphorylation	Promotion of the EMT, proliferation, migration, and invasion of lung cancer cells. Increase tumor size in vivo.	[83]
Exosomal let-7c-5p and miR-181b-5p	A549 cells	Unknown	Not experimentally tested	Inhibition of the EMT, migration, and invasion of BEAS-2B cells.	[84]
Exosomal miR-200	CAF	ZEB1	EMT-TF	Inhibition of the EMT, migration, and invasion of A549 and NCI-H460 cells.	[85]

EVs, extracellular vesicles. CSC, cancer stem cell. BALF, bronchoalveolar lavage fluid. CAF, cancer-associated fibroblast. SATB2, special AT-rich sequence-binding protein 2. FGFRL1, fibroblast growth factor receptor-like 1. FGF14, fibroblast growth factor 14. ZEB1, zinc finger E-box-binding homeobox 1. ERK, extracellular signal-regulated kinase. MEK, mitogen-activated protein kinase. mTOR, mammalian target of rapamycin. MMP, matrix metallopeptidase.

## 5. Potential Clinical Implications of miRNA Regulation of EMT in Lung Cancer

Understanding the role of miRNAs in regulating EMT in lung cancer holds significant clinical implications. This area of research can influence various aspects of lung cancer management, including miRNA-based therapies, risk assessments, diagnostics, and prognostic markers.

The reviewed studies indicate that directly targeting the miRNAs regulating EMT could be a viable therapeutic strategy. Evidence from in vivo experimentation highlights the critical role of miRNA regulation in EMT as a key driver of lung cancer progression. These studies indicate that specific miRNAs, such as miR-4739, miR-106a, miR-889-3p, miR-363-3p, exosomal miR-31-5p, and EVs-miR-1246b, are essential for the development of metastatic pulmonary nodules, bone metastasis, subcutaneous tumor growth, lung metastasis, and increased tumor size in vivo. These miRNAs have the best potential as candidates for therapeutic intervention as targeting them reverses such effects on lung cancer in vivo.

According to the reviewed studies, several other miRNAs acting as oncomiRs or TS miRNAs, such as miR-410, miR-6884-5p, miR-503, miR-188-5p, miR-203, and miR-146b, may have potential. However, these studies are based only on in vitro experimentation, and many provide limited information regarding the mechanisms involved in such regulation or fail to investigate them further. Much more research is needed on these miRNAs for potential clinical applications.

Another clinical implication is as diagnostic or prognosis markers in lung cancer. For instance, high levels of miR-4739 and miR-106a correlate with poor prognosis, while low levels of miR-6884-5p are associated with severe NSCLC progression. High levels of miR-4739 are also related to clinical staging and metastasis. High levels of EVs-miR-1246b in BALF are associated with malignant pulmonary nodules diagnosis. Furthermore, as EMT is linked to cancer progression, EMT-related miRNA can assist in stratifying patients based on their risk of metastasis, potential drug resistance, and overall prognosis. Therefore, understanding the underlying mechanism of the miRNA regulation of EMT in lung cancer could also help to address challenges such as detection, metastasis, and personalized therapy. However, as the recent literature showed, further research into EMT-related miRNAs is needed at this field stage.

## 6. Conclusions

microRNAs (miRNAs) are crucial in regulating the EMT, a process essential for cancer metastasis. EMT is essential in the early stages of cancer progression, as it allows cancer cells to break away from the primary tumor, invade surrounding tissues, and metastasize to other organs. In lung cancer, both endogenous miRNAs and those carried by EVs/exosomes play significant roles in advancing lung cancer by regulating the EMT process. Indeed, recently published studies show evidence of miRNAs’ significant role in lung cancer progression by regulating the EMT, as various miRNAs promote or inhibit metastasis and tumor growth in in vivo models (see Table 1 and Table 2). Elucidating the underlying mechanisms of these miRNAs (miR-4739, miR-106a, miR-889-3p, miR-363-3p, exosomal miR-31-5p, and EVs-miR-1246b) would provide a better understanding of the complex causes of lung cancer pathogenesis and progression and also identify potential therapy targets. The literature review also revealed that several recent studies on miRNAs regulating EMT in lung cancer have limited information regarding the mechanisms involved in such regulation. Many failed to investigate further a potential pathway involved in the miRNA/target gene regulating the EMT. Therefore, more research needs to be performed in this area to dissect the molecular mechanisms involved in miRNAs’ regulation of EMT. At the same time, it is crucial to find evidence of the critical role of miRNAs/EMT in metastasis and tumor growth using in vivo models to have a potential clinical application in therapy.

## Data Availability

This study did not create or analyze new data, and data sharing does not apply to this article.
